# The influences and neural correlates of past and present during gambling in humans

**DOI:** 10.1038/s41598-017-16862-9

**Published:** 2017-12-07

**Authors:** Pierre Sacré, Sandya Subramanian, Matthew S. D. Kerr, Kevin Kahn, Matthew A. Johnson, Juan Bulacio, Jorge A. González-Martínez, Sridevi V. Sarma, John T. Gale

**Affiliations:** 1 Institute for Computational Medicine, Department of Biomedical Engineering, The Johns Hopkins University, Baltimore, Maryland 21218 USA; 2 Epilepsy Center, Neurological Institute, Cleveland Clinic, Cleveland, Ohio 44195 USA; 3 Department of Neurosurgery, Emory University, Atlanta, Georgia 30322 USA

## Abstract

During financial decision-making tasks, humans often make “rational” decisions, where they maximize expected reward. However, this rationality may compete with a bias that reflects past outcomes. That is, if one just lost money or won money, this may impact future decisions. It is unclear how past outcomes influence future decisions in humans, and how neural circuits encode present and past information. In this study, six human subjects performed a financial decision-making task while we recorded local field potentials from multiple brain structures. We constructed a model for each subject characterizing bets on each trial as a function of present and past information. The models suggest that some patients are more influenced by previous trial outcomes (*i*.*e*., previous return and risk) than others who stick to more fixed decision strategies. In addition, past return and present risk modulated with the activity in the cuneus; while present return and past risk modulated with the activity in the superior temporal gyrus and the angular gyrus, respectively. Our findings suggest that these structures play a role in decision-making beyond their classical functions by incorporating predictions and risks in humans’ decision strategy, and provide new insight into how humans link their internal biases to decisions.

## Introduction

Decision-making links cognition to behavior and is a key driver of human personality, fundamental for survival, and essential for our ability to learn and adapt. Often, decisions are made under uncertainty, where choices are made given expected consequences^1^. Such decisions are thought to be “rational” when the subject is optimizing some criteria. For example, when gambling, subjects may wish to maximize their expected reward. However, it is well-established that humans often are influenced by internal biases such as preferences and emotions based on past experiences^2–4^. In fact, patients with psychiatric disorders frequently have alterations in decision-making, which stem from dysfunction of neural circuits that produce different cognitive and emotional symptoms affecting decision-making^5^.

Little is known about such disorders because the function of neural circuits involved in decision-making in humans is largely uncharted, severely limiting understanding of mechanistic changes underlying disruption associated with age or psychiatric diseases. In order to understand human decision-making, accessing several brain structures involved in decision-making and measuring their electrical activities at the millisecond resolution would be ideal. Typically, access to the human brain has been limited to a few case studies wherein subjects have lesions in a particular structure such as the orbitofrontal cortex (OFC)^5^, or studies where Positron Emission Tomography (PET) and Functional Magnetic Resonance Imaging (fMRI) are used to measure activity in several healthy subjects during decision-making^6^. Both of these approaches have limitations. Lesions don’t provide actual neural data to ascertain the brain’s role during behavior, and PET and fMRI provide correlates of neural activity but suffer from poor temporal resolution. PET resolution is on the order of minutes, fMRI is on the order of multiple seconds, while decisions are often made on the order of tenths of a second.

**Figure 1:**
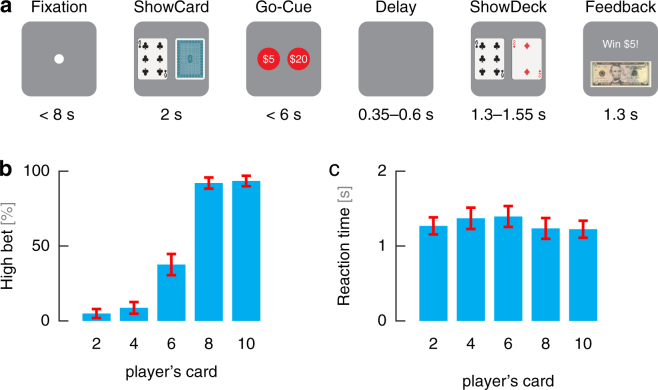
Gambling task and behavioral results. **(a)** Timeline of the behavioral task. After fixation, subjects were shown their card. Once the bets were shown, subjects selected one of the choices and then were shown the computer’s card following a delay. Feedback was provided afterwards by displaying the amount won or lost. **(b)** Average bet decisions across cards (±1 standard error of the mean). Subjects predominantly bet low for 2 and 4 cards and bet high for 8 and 10 cards. There was no dominant strategy for 6 cards, which had a 38% chance of eliciting a high bet. **(c)** Reaction times across cards (±1 standard error of the mean). Subjects reacted faster for cards whose rewards had lower variability. *Image copyrights:* The card images are reproduced without modification from Freedesignfile.com^49^ (license: https://creativecommons.org/licenses/by/3.0/). The United States five-dollar bill image is reproduced without modification from Wikipedia^50^.

In this study, simultaneous observations of electrical activity in multiple brain regions at timescales relevant to decision-making were obtained by placing several electrode contacts at these sources (Supplementary Fig. 1). Each contact recorded the activity of neuron populations at the local field potential level. Specifically, six human subjects implanted with StereoElectroEncephaloGraphy (SEEG) depth electrodes for clinical purposes performed a binary decision-making gambling task while local field potential neural activity was recorded (see subject information in Methods and Table 1). The task involved a game of high card where subjects won virtual money if their card was higher than the computer’s card as described previously^4^ (Fig. 1a). Subjects were informed that the deck of cards was infinite and only contains 2, 4, 6, 8 and 10 cards. On each trial, subjects had to decide to bet “high” ($20) or “low” ($5) on their card being higher than the hidden computer’s card drawn from the same deck.

**Table 1:** This table provides clinically relevant information on each of the subjects such as gender, age, duration of epilepsy (dur.), and the number of trials recorded for 6 cards as well as for all cards.

*Patient*	*Gender*	*Age* [*yr*.]	*Dur*. [*yr*.]	*Num*. *Trials* [6/*all*]	Epileptogenic zone	STG	Cu	AG
1	male	26	3	42/185	Hippo (L), FuG (L)	5 L	—	—
2	female	41	38	39/162	Hippo (R), TmP (R)	—	3 R	4 R
3	female	53	23	35/136	PCC (R), Ins (R), OFC (R)	8 R	—	—
4	female	60	8	33/172	TmP (L)	5 L/4 R	2 L	3 L/4 R
5	female	36	36	25/157	PrCu (R), IP sul (R), OcG (L), PO sul (L)	—	4 R	3 R
6	female	23	5	23/132	Hippo (L/R), ITG (R), IT sul (R)	2 R	2 R	5 R

AG, angular gyrus; Cu, cuneus; FuG, fusiform gyrus; Hippo, hippocampus; Ins, insula; IP sul, intraparietal sulcus; IT sul, inferior temporal sulcus; ITG, inferior temporal gyrus; OcG, occipital gyrus; OFC, orbitofrontal cortex; PCC, posterior cingulate cortex; PO sul, parieto-occipital sulcus; PrCu, precuneus; STG, superior temporal gyrus; TmP, temporal pole.

### Notations

In this paper, we use the following notations. The player’s card and computer’s card on trial *t* are denoted *PC*_*t*_ ∈ {2, 4, 6, 8, 10} and *CC*_*t*_ ∈ {2, 4, 6, 8, 10}, respectively. The binary betting decision is denoted by *Y*_*t*_ ∈ {0, 1}, where *Y*_*t*_ = 0 means that the participant bets low ($5) and *Y*_*t*_ = 1 means that the participant bets high ($20). The outcome of the card comparison is denoted *O*_*t*_ = sign(*PC*_*t*_ − *CC*_*t*_) ∈ {−1, 0, 1}, where −1 means loss, 0 means tie, and 1 means win. Random variables are usually denoted by an uppercase letter, while their realizations are denoted by a lowercase letter.

## Results

### Behavioral results

The optimal strategy for maximizing expected reward in this task is to bet high when dealt the 8 or 10 card and bet low when dealt the 2 or 4 card. Subject behavior closely followed this strategy, as they bet high ($20) 5%/9% of the time for the 2/4 cards and 92%/93% of the time for 8/10 cards, respectively (Fig. 1b). In contrast, for the 6 card, both betting options resulted in the same expected reward. The ambiguity of the decision on 6-card trials resulted in subjects taking longer to decide (Fig. 1c) as well as switching between both high and low bets. The average reaction time for 6-card trials was 1.39 s, while it was 1.27 s, 1.37 s, 1.24 s, and 1.22 s for 2, 4, 8, and 10 cards, respectively. For their decisions, subjects on average bet high 38% of the time for these 6-card trials. Although, these subjects are on average risk averse since they more often chose to risk less money on 6-card trials, they do not consistently bet low.

### Subject-specific probability models

**Figure 2:**
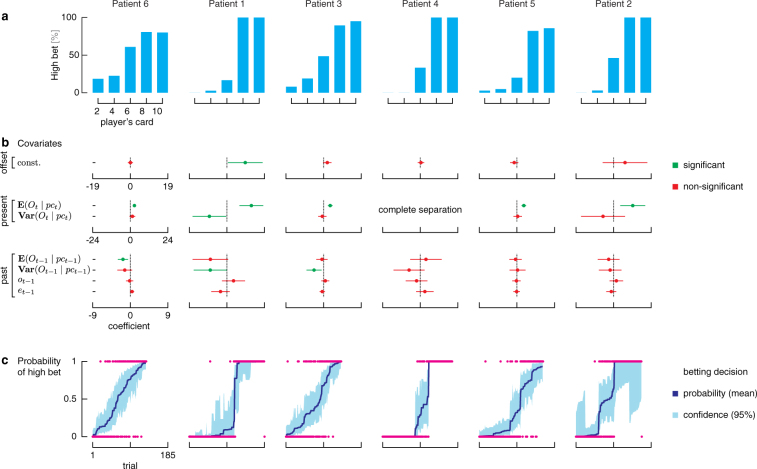
(**a**) Average bet decisions across cards. Subjects predominantly bet low for 2 and 4 cards and bet high for 8 and 10 cards. (**b**) The first panel shows the model coefficients and their 95% confidence bounds for each covariate for each patient model, also given in Supplementary Table 1. **(c)** The second panel overlays the estimated probability model ${\hat{p}}_{t}$ (blue curve) for each patient with the betting data (magenta dots), which are 0 for low bet and 1 for high bet. The trials are ordered from smallest ${\hat{p}}_{t}$ to largest ${\hat{p}}_{t}$ and the shaded region is the 95% confidence intervals for ${\hat{p}}_{t}\mathrm{.}$ *Remark*. The data for patient 4 shows a complete separation for 2, 4, 8, and 10-card trials. On these trials, the probability of high bets is equal to 0 for 2- and 4-card trials and it is equal to 1 for 8- and 10-card trials. Therefore, we fitted the model on 6-card trials only, excluding present covariates that are not useful or redundant with the constant term on 6-card trials.

Given that a decision must be made despite both decisions having the same expected reward, we hypothesized that decisions for these ambiguous trials (and trials where subjects did not maximize their expected reward) are influenced by past trial variables. To test our hypothesis, we constructed a behavioral model for each subject characterizing the probability of betting high on a trial as a function of (i) *present* covariates quantified by the expected outcome given the player’s card (**E**(*O*_*t*_|*pc*_*t*_)) and the variance of the outcome given the player’s card (**Var**(*O*_*t*_|*pc*_*t*_)), and (ii) *past* covariates quantified by the previous trial’s expected outcome given the player’s card (**E**(*O*_*t*_−_1_|*pc*_*t*_−_1_)), variance of outcome given the player’s card (**Var**(*O*_*t*−1_|*pc*_*t*−1_)), outcome (*o*_*t*−1_), and outcome-prediction error (*e*_*t*−1_ = *o*_*t*−1_−*po*_*t*−1_, where *po*_*t*_ is equal to −1[1−*y*_*t*_] + 1*y*_*t*_). These terms modulate the baseline probability of betting high on trial *t*, captured by the constant term. Parameters of the models were fit via maximum likelihood estimation methods (see Methods, Fig. 2, and Supplementary Table 1).

The present return was significant in models across all subjects and the present risk was significant in model of subject 1. In addition, one of the past components was also significant in three of the six subjects (subjects 1, 3, and 6). Subjects 1 and 3 were influenced by the previous trial’s risk (quantified by **Var**(*O*_*t*−1_|*pc*_*t*−1_)) and subject 6 was influenced by her previous trial’s return (quantified by **E**(*O*_*t*−1_|*pc*_*t*−1_)). This is not surprising when one observes the betting behavior as a function of previous trial parameters as illustrated in Supplementary Fig. 2. Subjects 1, 3, and 6 are clearly changing their betting strategies based on previous trial return and risk. For example, subject 1 is more likely to bet high on a 6-card trial if his previous trial was a low risk trial (*i.e*., previous card value is a 2 or 10); and subject 6 is more likely to bet high on any card if she just had a 2 card on her previous trial. Although previous outcome and outcome-prediction error seem to influence betting behavior on some subjects, there are not enough data samples to render significance in the models.

### Neural correlates

We searched for neural correlates of the two model components that were significant in at least one subject: (a) expected outcome and (b) variance of the outcome. For both components, we looked at the encoding of the information and the retrieval of the information. For the present encoding of the information, we searched for neural data at trial *t* modulating with the variable at trial *t* (correlate of the present variable in the present neural data) and we hypothesized that it happened right after the present player’s card was revealed. For the retrieval of past information, we searched for neural data at trial *t* modulating with the variable at trial *t* − 1 (correlate of the past variable in the present neural data) and we hypothesized that it happened right before the present player’s card was revealed. We conjectured that these components are being encoded in all subjects, while only half are influenced by them when they make their decisions. Therefore, we analyzed neural activity in all subjects.

To identify neural correlates of a given variable, we constructed spectrograms for each trial time-locked on a specific epoch for each brain region and hemisphere. Then, we performed a nonparametric cluster-based statistical test on spectrograms to identify modulation with the variable of interest^7^. This test naturally solves the problem of multiple comparisons (one comparison for each time-frequency windows) as described in methods. To approach this analysis with an unbiased viewpoint of the whole brain, we looked at every region that fulfilled the following requirements: (i) the region must have been recorded in at least three different subjects, and (ii) the regions must not be part of the clinically annotated epileptogenic zone for any subject. These requirements are motivated by the low sample size of our population. We report the most significant results for model covariates during encoding and retrieval after applying a false discovery rate (with *q* = 0.05) to solve the problem of multiple comparisons (one comparison for each brain regions).

#### Correlates of Return

**Figure 3:**
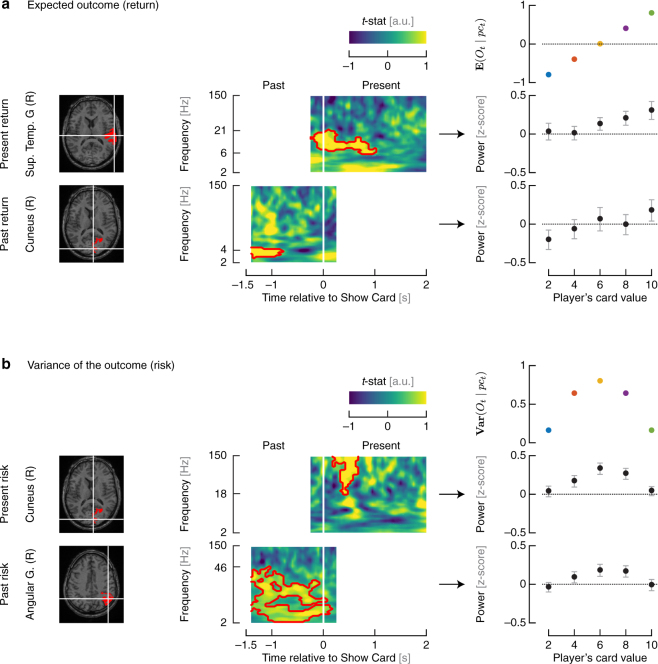
Neural correlates of encoding and retrieval for return and risk variables. **(a)** The neural activity in superior temporal gyrus (R) after the Show Card epoch of trial *t* modulates with the expected outcome at trial *t*. The neural activity in cuneus (R) before the Show Card epoch of trial *t* modulates with the expected outcome at trial *t* − 1. The time-frequency plot maps the *t*-statistic associated with the Pearson’s correlation between the variable and the neural data in each window. The red contour highlights the time-frequency cluster that shows a significant correlation (two-tailed test, *α* = 0.05). The average power in the cluster shows a modulation with the player’s card that is similar to the one of the variable of interest. Error bars represent ±1.96 standard errors from the mean. **(b)** The neural activity in cuneus (R) after the Show Card epoch of trial *t* modulates with the variance of the outcome at trial *t*. The neural activity in angular gyrus (R) before the Show Card epoch of trial *t* modulates with the variance of the outcome at trial *t* − 1.

Return can be quantified by the expected outcome given the player’s card. The expected outcome is a linear function of the player’s card regardless of the bet (as shown in Fig. 3a). For encoding of present return, we found that the superior temporal gyrus (R) has the most significant correlate as measured by its cluster (*p* = 0.0012). In particular, immediately *after* the subject sees his/her card, power in the 6–21 Hz band increases with the expected outcome given the player’s card on a given trial. Not surprisingly, the average power in the superior temporal gyrus (R) cluster follows a linear trend as a function of the player’s card. For retrieval of past return, we found that the cuneus (R) has the most significant correlate as measured by its cluster (*p* = 0.0012). In particular, about one second *before* the player’s card of the current trial is shown, power in the 2–4 Hz band increases with the expected outcome on the previous trial.

#### Correlates of Risk

Risk can be quantified by the variance of the outcome given the player’s card. The variance of the outcome is a concave function of the player’s card regardless of the bet (as shown in Fig. 3b). For encoding of present risk, we found that the cuneus (R) has the most significant correlate as measured by its cluster (*p* = 0.0002). In particular, immediately *after* the subject sees his/her card, power in the 18–150 Hz band increases with the variance of the outcome given the payer’s card on a given trial. Not surprisingly, the average power in the cuneus (R) cluster after the player’s card is shown also follows the same concave shape. For retrieval of past risk, we found that the angular gyrus (R) has the most significant correlate as measured by its cluster (*p* = 0.0002). In particular, during one second *before* the player’s card of the current trial is shown, power in the 2–46 Hz band increases with the variance of the outcome on the previous trial.

## Discussion

In this study, we hypothesized that past trial outcomes bias subjects’ gambling decisions, especially on trials where uncertainty is the largest and where present information cannot guide subjects (*e.g*., 6 card trials). To test this hypothesis, we constructed subject-specific models for the probability of betting high on a given trial as a function of the present return and risk, and past information including previous trial’s return, risk, outcome, and outcome-prediction error. The models showed that one or more past covariates improved model fit in 3 of the 6 subjects.

For the subjects influenced by past covariates (1, 3, and 6), there were clear changes in betting strategies. That is, they did not always bet high on 8 and 10 and low on 2 and 4, and their bets on 6 card trials could not be captured by a simple biased coin flip model (constant probability of betting high on 6 cards). Rather, they sometimes bet high on 6 cards and sometimes low on 6 cards, which can be predicted by past outcomes. In contrast, for other subjects, all past covariates were insignificant in the model. Although our study cohort is small, our results suggest that humans range the entire spectrum as decision makers from past-dependent to past-independent.

We then looked for neural correlates of encoding (present) and retrieval (past) of each model component that was significant in at least one subject’s model. Since return and risk significantly influence betting behavior, these components must be encoded in the brain right after the player’s card is shown in a trial and then must be retrieved during the next trial to weigh into the decision on the next trial.

### Superior temporal gyrus

We found that alpha and beta-band power in the superior temporal gyrus (R) encodes the present return or expected outcome once the subject sees his/her card. The superior temporal gyrus is one of the three gyri of the temporal lobe. It is bounded by the lateral sulcus above and the superior temporal sulcus below. It is mainly known to be responsible for processing of both object- and space-related information^8^, but has been shown to be involved in integrating previous actions and outcomes into one’s decision-making strategy^9^. The role of superior temporal gyrus in non-speech related processing has been described as a correlate of temporal action planning^10^, judgment tasks^11^, and processing complex configurations of symbolic information^12^. Superior temporal gyrus activation has also been linked to higher semantic associations^13^, context dependent processing^14^, and with detecting the degree of predictability in a variably random sequence of stimulus presentations^15^. Taken together, our study and past works suggest that the superior temporal gyrus provides predictive information that occurs over time in order to identify, process, and/or prepare for actions.

### Cuneus

We found that gamma and high gamma-band power in the cuneus (R) encodes present risk or variance of outcome once the subject sees his/her card, and that delta power in the cuneus may be involved in retrieval of past return at the beginning of the current trial before the subject sees his/her card. The cuneus is a small lobe within the occipital lobe. It is bounded anteriorly by the parieto-occipital sulcus and inferiorly by the calcarine sulcus. It is mainly known to be involved in basic visual processing^16^, but several fMRI studies have shown that cuneus is involved in decision-making. Pathologic gamblers have higher fMRI bold activity in the dorsal visual processing stream including the cuneus relative to controls^17^, and gray matter volume in the cuneus is associated with better inhibitory control in bipolar depression patients^18^. In addition, adolescents with internet gambling addiction show high fMRI cuneus activity during go/no-go tasks^19^. Furthermore, fMRI activity in the cuneus appears to be higher when subjects chase losses when gambling but is lower when they quit gambling^20^. Other studies show cuneus fMRI activity correlating with value, probability of winning, and saliency^21,22^. Past studies suggest that the cuneus may indeed be involved in calculating risk and also return, but have not had the temporal resolution in neural data that SEEG recordings provide to be more precise about fast oscillations and dynamics of electrical activity in the cuneus during risky decision-making. Taken together, our study and past works strongly suggest that cuneus impacts human decision-making and not just basic visual processing.

### Angular gyrus

We found that power between 2–46 Hz in the angular gyrus (R) retrieves the past trial’s risk value. The angular gyrus is a brain region in the parietal lobe that lies near the superior edge of the temporal lobe and immediately posterior to the supramarginal gyrus. It is mainly known to be involved in complex language functions, but previous fMRI studies have also linked angular gyrus to decision-making^23–25^. In particular, the activation of angular gyrus reflects the probability^22,26,27^ and variance^28^ of potential outcomes. The angular gyrus has also been linked to the orientation of visuospatial attention^29,30^. The attentional focus can influence both the processing of a decision and the choice being made^31–33^. The attentional effort can also be driven by the decision difficulty^34^. Furthermore, reward-associated features of visual stimuli can attract and capture attention, even when they are no longer relevant^35–37^. This relationship might be particularly relevant to the understanding of the role of the angular gyrus in retrieving past outcome information in decision-making. Taken together, our study and past works suggest that the angular gyrus might be involved in guiding attention within the visual representation of decision information, which then enables retrieval of past outcomes relevant to current choice.

### Conclusion

In conclusion, SEEG recordings offer a unique opportunity to understand spatio-temporal dynamics during decision-making at a millisecond resolution, and our task allows us to examine how humans make decisions in the face of variable uncertainty. The neural correlates found here warrant further examination of the superior temporal gyrus, the cuneus, and the angular gyrus, and their role in decision-making.

## Methods

### Subjects

Patients with medically intractable epilepsy increasingly undergo StereoElectroEncephaloGraphy (SEEG) recording in order to localize and resect the seizure focus. In this study, aside from the behavioral experiments no alterations were made to the patients’ clinical care, including the placement of the electrodes^38^. A total of six subjects volunteered to perform the task.

Subjects were implanted with 9 to 13 depth electrodes. Implantation was performed using robot-assisted surgery along with co-registered MRIs and CT scans, using methods previously described^39^. Once inserted, SEEG electrophysiological data were acquired using a Nihon Kohden 1200 EEG diagnostic and monitoring system (Nihon Kohden America, USA) at a sampling rate of 1 or 2 kHz. Behavioral task event marker were written to and simultaneously with the electrophysiological data using the MonkeyLogic Matlab toolbox^40^.

Details on these patients and the number of contacts in each reported structure for each subject are noted in Table 1.

It is important to acknowledge standard concerns in analyzing data from epileptic patients. First, patients are often on medication, which might affect the neurophysiology of the brain. For clinical purposes, patients were kept off of their anti-seizure medication for their entire stay in the hospital, so these effects would be minimized. Secondly, actual seizures might impact the neurophysiology around the seizure focus. Human epilepsy recordings are taken to localize the seizure focus, so overlap is possible between seizure focus and areas recorded. In our cohort, the seizure foci did not include the superior temporal gyrus, the cuneus, and the angular gyrus.

#### Ethics statement

All experimental protocols were approved by the Cleveland Clinic Institutional Review Board. Experiments and methods were performed in accordance with the guidelines and regulations of the Cleveland Clinic Institutional Review Board. All subjects volunteered and provided informed consent in accordance with the guidelines of the Cleveland Clinic Institutional Review Board.

### Stereoelectroencephalography

Stereotactically implanted depth electrodes (SEEG) is a growing brain imaging modality at Cleveland Clinic^41^. It is a technique and method that was developed in France^42,43^, and that is becoming more prevalent at epilepsy centers in the US. In routine placement of depth electrodes, burr-holes (15–35 mm in diameter) are required for safe visualization of cortical vessels, and therefore only a small number of electrodes are placed. SEEG placement, however, uses small drill holes (1.8 mm in diameter), allowing many electrodes to be safely inserted. In addition, SEEG electrode placements are guided by pre-implantation hypotheses of epileptic networks localization. SEEG provides a more complete coverage of the brain, from lateral, intermediate and/or deep structures in a three-dimensional arrangement recorded over hundreds of channels (Supplementary Fig. 1). Using strict techniques, this procedure is safe and minimally invasive: only 1/1176 implantations last year resulted in an asymptomatic intracranial hemorrhage. The rate of complications in SEEG implantations is less than 1 %^41,44^. Among all methods of invasive monitoring, SEEG is the least morbid^45^.

### Gambling task

The behavioral task was performed in the subject’s Epilepsy Monitoring Unit room. The task was presented via a computer screen and the subject interacted with the task using an InMotion2 robotic manipulandum (Interactive Motion Technologies, USA). The manipulandum is controlled by the subject’s hand and allows for 2D planar motion which is translated directly to the position of a cursor onscreen.

The gambling task (Fig. 1a) is based on a simple game of high card where subjects would win virtual money if their card beat the computer’s card as previously described^4,46,47^. The subjects are told that (i) the deck of cards is infinite, (ii) the deck only consists of five different cards (2, 4, 6, 8, or 10), and (iii) each card is equally likely to occur on each draw. In the beginning of each trial, the subject controlled a cursor—via a planar manipulandum—to a fixation target. During fixation, subjects must center the cursor in less than 8 seconds. Once centered, the subject is shown his card (2, 4, 6, 8, or 10) for a duration of 2 seconds. The player’s card is randomly chosen with equal distribution. The computer’s card is initially hidden. The screen then shows the two possible choices: a high bet ($20) or a low bet ($5). The subject has 6 seconds to select one with his cursor. Following selection the computer’s card, which follows the same distribution, is revealed. If the computer’s card is larger than the player’s card, then the subject loses the amount he bets. If the computer’s card is smaller than the player’s card, then the subject wins the amount he bets. If the two cards are equal then no virtual money is won or lost. The final screen depicts the amount won or lost. See Fig. 1a for timeline of task trial.

### Modeling betting behavior

For any trial *t*, the probability of betting high is denoted by *p*_*t*_ = **prob**(*Y*_*t*_ = 1) ∈ [0, 1]. This probability *p*_*t*_ is modeled as a function of an *offset*, *present* covariates ${x}_{t}^{{\rm{p}}{\rm{r}}{\rm{e}}{\rm{s}}}$, and *past* covariates ${x}_{t}^{{\rm{p}}{\rm{a}}{\rm{s}}{\rm{t}}}$, that is,

1 $${p}_{t}=\frac{\exp ({\beta }^{T}{x}_{t})}{1+\exp ({\beta }^{T}{x}_{t})},$$

where

$${x}_{t}^{T}=[1,{x}_{t}^{{\rm{p}}{\rm{r}}{\rm{e}}{\rm{s}}T},{x}_{t}^{{\rm{p}}{\rm{a}}{\rm{s}}{\rm{t}}T}]\quad {\rm{a}}{\rm{n}}{\rm{d}}\quad {\beta }^{T}=[{\beta }^{0T},{\beta }^{{\rm{p}}{\rm{r}}{\rm{e}}{\rm{s}}T},{\beta }^{{\rm{p}}{\rm{a}}{\rm{s}}{\rm{t}}T}].$$

The *present* covariates ${x}_{t}^{{\rm{p}}{\rm{r}}{\rm{e}}{\rm{s}}}$ consist of the expected outcome given the player’s card (**E**(*O*_*t*_|*pc*_*t*_)) and the variance of the outcome given the player’s card (**Var**(*O*_*t*_|*pc*_*t*_)). The *past* covariates ${x}_{t}^{{\rm{p}}{\rm{a}}{\rm{s}}{\rm{t}}}$ consist of four statistics characterizing the previous trial including the previous expected outcome (**E**(*O*_*t*−1_|*pc*_*t*−1_)), the previous variance of the outcome (**Var**(*O*_*t*−1_|*pc*_*t*−1_)), the previous outcome (*o*_*t*−1_), and the previous outcome-prediction error (*e*_*t*−1_). Here, the outcome-prediction error *e*_*t*_ is the difference between the outcome *o*_*t*_ and the predicted outcome *po*_*t*_ based on the bet, that is,

$${e}_{t}={o}_{t}-p{o}_{t},$$

where the predicted outcome *po*_*t*_ is defined as follows

$$p{o}_{t}=-1\,[1-{y}_{t}]+1\,{y}_{t}=\{\begin{array}{ll}1 & {\rm{if}}\,{y}_{t}=1,\\ 1 & {\rm{if}}\,{y}_{t}=0.\end{array}$$

This definition means that the player predicts to win when betting high (*i.e*., *po*_*t*_ = 1 if *y*_*t*_ = 1) and predicts to lose when betting low (*i.e*., *po*_*t*_ = −1 if *y*_*t*_ = 0).

#### Intuition about the model structure and its estimation

To construct a model of the form (1) for each subject, we estimated the vector *β* by maximizing the data likelihood function. The structure of model (1) was chosen for the following reasons. First, Eq. (1) ensures that the probability of betting high is a value restricted to be between 0 and 1. Second, Eq. (1) is a Generalized Linear Model (GLM) for Bernoulli observations (bets) which ensures that the data likelihood function is concave in the unknown parameters *β*. This is desirable to ensure a unique global maximum and one can compute the maximum likelihood estimate efficiently^48^.

### Neural correlates data analysis

All electrophysiological and behavioral analyses were conducted offline using custom MATLAB® scripts. To search for neural correlates of the model components (*e.g*., expected outcome, variance of outcome) in the multiple brain regions (looking at each hemisphere separately), we examined the modulation of the neural responses in the spectral domain with a specified task variable during a specific epoch in the trial by means of a non-parametric cluster statistic^7^.

#### Spectral analysis

Oscillatory power was calculated using continuous wavelet transform (logarithmic scale vector ranging 2–150 Hz and a complex Morlet wavelet with ***ω***_0_ = 6). Then, we binned the instantaneous power spectral density into 100-ms time windows spaced every 50 ms (50% overlap) and averaged the instantaneous power spectral density over each time window. Each 100-ms time window is marked with the time that corresponds to the end of the temporal window. Afterwards, each frequency bin’s power was log normalized based on the power across the entire recording session by fitting the log of the power in each frequency bin to a standard normal distribution.

#### Nonparametric cluster test

We used a nonparameteric cluster test to find neural correlates^7^. This test leverages the dependency between adjacent time-frequency windows in order to avoid over-penalizing with multiple comparison corrections. The main idea of this test is to build clusters (that is, set of adjacent time-frequency windows whose activity statistically significantly modulates with the design variable of interest) and to evaluate their significance by comparing a test statistic for the observed clusters to a null distribution generated by shuffling the label 10000 times between trials within each subject. To build clusters, a Pearson’s correlation *r* between the neural data in each time-frequency window and the shuffled variable labels was calculated. For each time-frequency window, the correlation *r* was transformed to a *t*-statistic as follows $t=r\sqrt{(n-2)/(1-{r}^{2})}$ and a *p*-value was assigned. Clusters were formed by grouping windows with significant correlations of the same sign that were adjacent in either time or frequency (using a two-tailed test with a significance level *α* = 0.05). The test statistic for each cluster was calculated by taking the sum across each window in the cluster of the difference between the *t*-statistic and the critical value corresponding to the intermediary threshold. This prioritizes clusters that both have strong differences as well as large sizes. The observed cluster statistic was obtained with the original labels. In turn, it was then compared against this null distribution of cluster statistics in order to obtain the final *p*-value of the test.

#### False discovery rate

We corrected the significance threshold using a false discovery rate with *q* = 0.05 in order to solve the problem of multiple comparisons (one comparison for each brain region).

## Electronic supplementary material

Supplementary Information

## References

[1] Knight, F. H. *Risk*, *uncertainty and profit*. Hart, Schaffner and Marx’s series (Houghton Mifflin Company, New York, NY, 1921).

[2] Kacelnik A, Bateson M (1997). Risk-sensitivity: crossroads for theories of decision-making. Trends Cogn. Sci..

[3] Kim H, Choi M-J, Jang I-J (2011). Lateral OFC activity predicts decision bias due to first impressions during ultimatum games. J. Cognitive Neurosci..

[4] Sacré P (2016). Lucky rhythms in orbitofrontal cortex bias gambling decisions in humans. Sci. Rep..

[5] Bechara A (2004). The role of emotion in decision-making: Evidence from neurological patients with orbitofrontal damage. Brain Cogn..

[6] Logothetis NK (2008). What we can do and what we cannot do with fMRI. Nature.

[7] Maris E, Oostenveld R (2007). Nonparametric statistical testing of EEG- and MEG-data. J. Neurosci. Methods.

[8] Karnath H-O (2001). New insights into the functions of the superior temporal cortex. Nat. Rev. Neurosci..

[9] Paulus MP, Feinstein JS, Leland D, Simmons AN (2005). Superior temporal gyrus and insula provide response and outcome-dependent information during assessment and action selection in a decision-making situation. NeuroImage.

[10] Kircher TT, Brammer MJ, Levelt W, Bartels M, McGuire PK (2004). Pausing for thought: engagement of left temporal cortex during pauses in speech. NeuroImage.

[11] Luo Q (2003). The neural substrate of analogical reasoning: an fMRI study. Cogn. Brain Res..

[12] Grossman M (2002). Sentence processing strategies in healthy seniors with poor comprehension: An fMRI study. Brain Lang..

[13] Jessen F (1999). Activation of human language processing brain regions after the presentation of random letter strings demonstrated with event-related functional magnetic resonance imaging. Neurosci. Lett..

[14] Opitz B, Mecklinger A, Friederici A, von Cramon D (1999). The functional neuroanatomy of novelty processing: Integrating ERP and fMRI results. Cereb. Cortex.

[15] Bischoff-Grethe A, Proper SM, Mao H, Daniels KA, Berns GS (2000). Conscious and unconscious processing of nonverbal predictability in Wernicke’s area. J. Neurosci..

[16] Vanni S, Tanskanen T, Seppä M, Uutela K, Hari R (2001). Coinciding early activation of the human primary visual cortex and anteromedial cuneus. Proc. Natl. Acad. Sci. USA.

[17] Crockford DN, Goodyear B, Edwards J, Quickfall J, el Guebaly N (2005). Cue-induced brain activity in pathological gamblers. Biol. Psychiatry.

[18] Haldane M, Cunningham G, Androutsos C, Frangou S (2008). Structural brain correlates of response inhibition in Bipolar Disorder I. J. Psychopharmacol..

[19] Ding W-n (2014). Trait impulsivity and impaired prefrontal impulse inhibition function in adolescents with internet gaming addiction revealed by a Go/No-Go fMRI study. Behav. Brain Funct..

[20] Campbell-Meiklejohn DK, Woolrich MW, Passingham RE, Rogers RD (2008). Knowing when to stop: The brain mechanisms of chasing losses. Biol. Psychiatry.

[21] Litt A, Plassmann H, Shiv B, Rangel A (2011). Dissociating valuation and saliency signals during decision-making. Cereb. Cortex.

[22] Studer B, Apergis-Schoute A, Robbins T, Clark L (2012). What are the odds? The neural correlates of active choice during gambling. Front. Neurosci..

[23] Vickery TJ, Jiang YV (2009). Inferior parietal lobule supports decision making under uncertainty in humans. Cereb. Cortex.

[24] Labudda K (2008). Neural correlates of decision making with explicit information about probabilities and incentives in elderly healthy subjects. Exp. Brain Res..

[25] Ernst M (2004). Choice selection and reward anticipation: an fMRI study. Neuropsychologia.

[26] Berns GS, Capra CM, Chappelow J, Moore S, Noussair C (2008). Nonlinear neurobiological probability weighting functions for aversive outcomes. NeuroImage.

[27] Bach DR, Hulme O, Penny WD, Dolan RJ (2011). The known unknowns: Neural representation of second-order uncertainty, and ambiguity. J. Neurosci..

[28] Symmonds M, Wright ND, Bach DR, Dolan RJ (2011). Deconstructing risk: Separable encoding of variance and skewness in the brain. NeuroImage.

[29] Chechlacz M, Rotshtein P, Humphreys G (2012). Neuroanatomical dissections of unilateral visual neglect symptoms: ALE meta-analysis of lesion-symptom mapping. Front. Hum. Neurosci..

[30] Rushworth M, Taylor P (2006). TMS in the parietal cortex: Updating representations for attention and action. Neuropsychologia.

[31] Krajbich I, Armel C, Rangel A (2010). Visual fixations and the computation and comparison of value in simple choice. Nat. Neurosci..

[32] Kovach C, Sutterer M, Rushia S, Teriakidis A, Jenison R (2014). Two systems drive attention to rewards. Front. Psychol..

[33] Armel, K. C., Beaumel, A. &Rangel, A. Biasing simple choices by manipulating relative visual attention. *Judgm. Decis. Mak*. **3**, 396–403. http://journal.sjdm.org/8319/jdm8319.pdf (2008).

[34] Philiastides MG, Ratcliff R, Sajda P (2006). Neural representation of task difficulty and decision making during perceptual categorization: A timing diagram. J. Neurosci..

[35] Hickey C, Chelazzi L, Theeuwes J (2010). Reward changes salience in human vision via the anterior cingulate. J. Neurosci..

[36] Chelazzi L, Perlato A, Santandrea E, Libera CD (2013). Rewards teach visual selective attention. Vision Res..

[37] Anderson BA, Laurent PA, Yantis S (2011). Value-driven attentional capture. Proc. Natl. Acad. Sci. USA.

[38] Johnson, M. A. *et al*. Performing behavioral tasks in subjects with intracranial electrodes. *J. Vis. Exp*., e51947; 10.3791/51947 (2014). 10.3791/51947 PMC4672964 25349952

[39] González-Martínez J (2016). Technique, results, and complications related to robot-assisted stereoelectroencephalography. Neurosurgery.

[40] Asaad WF, Eskandar EN (2008). A flexible software tool for temporally-precise behavioral control in Matlab. J. Neurosci. Methods.

[41] González-Martínez J (2014). Stereotactic placement of depth electrodes in medically intractable epilepsy. J. Neurosurg..

[42] Talairach, J. & Szikla, G. Application of stereotactic concepts to the surgery of epilepsy. In Gillingham, F., Gybels, J., Hitchcock, E., Rossi, G. &Szikla, G. (eds.) *Advances in**Stereotactic and Functional Neurosurgery 4*, vol. 30 of*Acta Neurochirurgica Supplementum*, 35–54 (Springer Vienna, 1980). 10.1007/978-3-7091-8592-6_5 7008525

[43] Talairach J, Tournoux P, Musolino A, Missir O (1992). Stereotaxic exploration in frontal epilepsy. Adv. Neurol..

[44] Cardinale F, Lo Russo G (2013). Stereo-electroencephalography safety and effectiveness: Some more reasons in favor of epilepsy surgery. Epilepsia.

[45] Mullin JP (2016). Is SEEG safe? A systematic review and meta-analysis of stereo-electroencephalography–related complications. Epilepsia.

[46] Gale, J. T., Martinez-Rubio, C., Sheth, S. A. & Eskandar, E. N. Intra-operative behavioral tasks in awake humans undergoing deep brain stimulation surgery. *J. Vis. Exp*., e2156; 10.3791/2156 (2011). 10.3791/2156 PMC3182655 21248697

[47] Patel SR (2012). Single-neuron responses in the human nucleus accumbens during a financial decision-making task. J. Neurosci..

[48] McCullagh, P. & Nelder, J. A. *Generalized linear models* (Chapman & Hall/CRC, 1989), 2nd edn.

[49] Freedesignfile.com. Different playing card vector graphic 05.http://freedesignfile.com/22764-different-playing-card-vector-graphic -05/. Last accessed on April 28, 2017 (2017).

[50] Slick-o-bot. US $5 series 2006 obverse. https://commons.wikimedia.org/wiki/File:US_$5_Series_2006_obverse.jpg. Last accessed on April 28, 2017 (2013).

