# Supplementary material for: The influences and neural correlates of past and present during gambling in humans
Source: Sci Rep. 2017 Dec 7;7:17111. doi: 10.1038/s41598-017-16862-9 (PMC5719351; doi:10.1038/s41598-017-16862-9)
Supplement: Supplementary file 1 — Supplementary Information [file 41598_2017_16862_MOESM1_ESM.pdf]

# Supplementary Information: The influences and neural correlates of past and present during gambling in humans

**Pierre Sacré[1,*,†], Sandya Subramanian[1,†], Matthew S. D. Kerr[1], Kevin Kahn[1], Matthew A. Johnson[2], Juan Bulacio[2], Jorge A. González-Martínez[2], Sridevi V. Sarma[1,*,‡], and John T. Gale[3,‡]**

[1]Institute for Computational Medicine, Department of Biomedical Engineering, The Johns Hopkins University, Baltimore, Maryland 21218, USA.
[2]Epilepsy Center, Neurological Institute, Cleveland Clinic, Cleveland, Ohio 44195, USA.
[3]Department of Neurosurgery, Emory University, Atlanta, Georgia 30322, USA.
[*]Correspondence should be addressed to P.S. (p.sacre@jhu.edu) or S.V.S. (ssarma2@jhu.edu).
[†]These authors contributed equally to this work.
[‡]These authors jointly supervised this work.

The Supplementary Information contains:

- **Supplementary Fig. 1**;
- **Supplementary Fig. 2**;
- **Supplementary Table 1**.

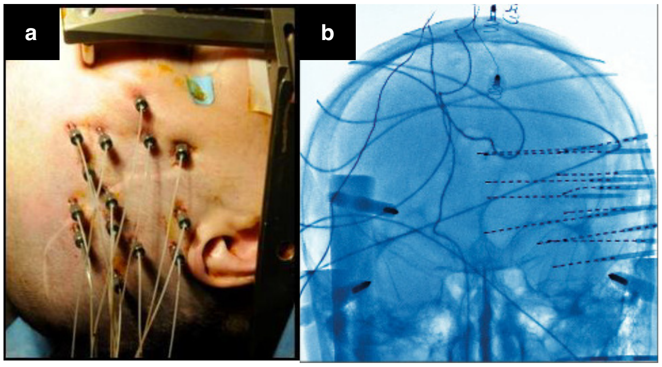


**Supplementary Figure 1.** Imaging fusion and placement of multiple electrodes using the SEEG method. **(a)** The side-view photograph shows 14 electrodes at the skin surface. **(b)** A fluoroscopy image of an SEEG-implanted subject (coronal view with eye forward) shows the precise parallel placement, with tips terminating at the midline or dural surface.

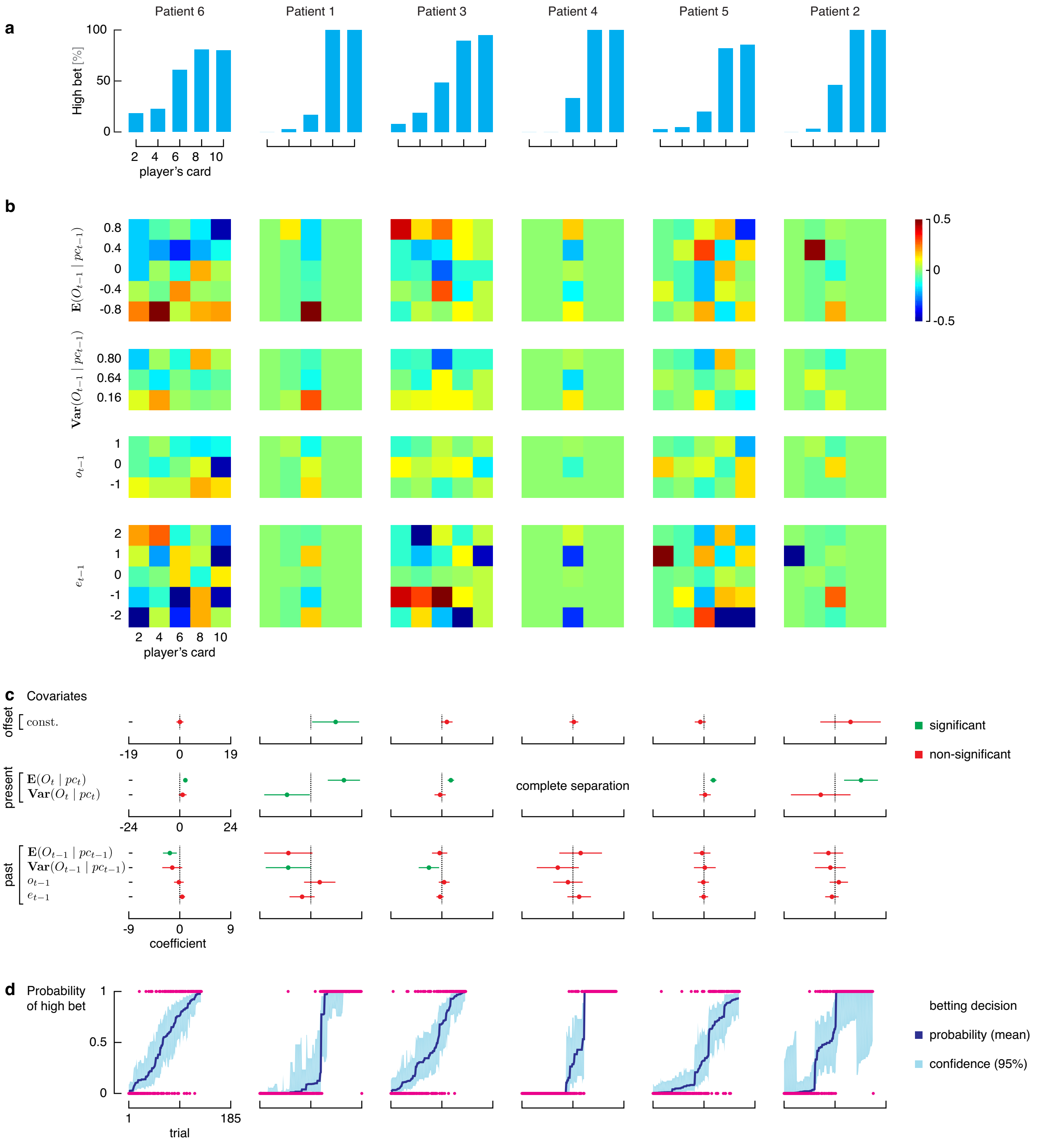


**Supplementary Figure 2.** **(a)** This panel represents the average bet decisions across cards. Subjects predominantly bet low for 2 and 4 cards and bet high for 8 and 10 cards. **(b)** This panel (4 rows) represents betting behavior as a function of previous trial parameters. The abscissa represents the card value (2, 4, 6, 8, 10) and the ordinate represents the past outcome variable value. The heatmap represents the fraction above (positive value) or below (negative value) baseline of the percentage of high bets. For example, consider Patient 3 (3rd column) and Previous prediction error (4th row). For Patient 3, when she experienced trials where her prediction error was $-1$, and then each of these trials was followed by a 2, 4, or 6 card, then she was more likely to bet high as indicated by red colors in the heatmap plot. The patients are ordered from more biased by previous outcomes to less biased as you move from left to right. That is, present-and-past patients each had at least one previous outcome variable significant in their model, while present-only patients (3 rightmost patients) did not. **(c)** This panel show the model coefficients and their 95 % confidence bounds for each covariate for each patient model, also given in **Supplementary Table 1**. Note that the top two coefficients are the present components and the remaining four are the past components. **(d)** This panel overlays the probability model, $\hat{p}_t$, (blue curve) for each patient with the betting data which are 0 for low bet and 1 for high bet (magenta). The trials are ordered from smallest $\hat{p}_t$ to largest $\hat{p}_t$ and the grey shaded region is the 95 % confidence intervals for $\hat{p}_t$.

**Supplementary Table 1.** This table provides the parameter values for the model coefficients that maximize the likelihood of observing all trials of each subject. Coefficients with significant $p$-values ($< 0.05$) are in blue.

| | | *present* | | *past* | | | |
|---|---|---|---|---|---|---|---|
| *Patient* | const. | $\mathbf{E}(O_t \mid pc_t)$ | $\mathbf{Var}(O_t \mid pc_t)$ | $\mathbf{E}(O_{t-1} \mid pc_{t-1})$ | $\mathbf{Var}(O_{t_1} \mid pc_{t-1})$ | $o_{t-1}$ | $e_{t-1}$ |
| 1 | **9.25** | **15.58** | **−11.21** | −3.96 | **−4.00** | 1.57 | −1.54 |
| 2 | 5.78 | **12.23** | −6.74 | −1.17 | −0.83 | 0.67 | −0.54 |
| 3 | 1.86 | **4.22** | −0.83 | −0.39 | **−2.29** | 0.40 | −0.32 |
| 4 | 0.35 | – | – | 1.36 | −2.68 | −0.88 | 1.10 |
| 5 | −1.37 | **4.39** | 0.49 | −0.33 | 0.15 | −0.12 | −0.06 |
| 6 | 0.06 | **2.64** | 1.34 | **−1.75** | −1.33 | −0.16 | 0.43 |
